# ‘Masking’ emotions: doctor–patient communication in the era of COVID-19

**DOI:** 10.1136/postgradmedj-2020-138444

**Published:** 2020-07-01

**Authors:** Keshinie Samarasekara

**Affiliations:** University Medical Unit, National Hospital of Sri Lanka, Colombo, Sri Lanka

Effective communication is a cornerstone of a healthy doctor–patient relationship in almost any clinical setting. As physicians, we find that our empathetic communication is of paramount importance to our patients and may even be therapeutic to some. A significant proportion of this communication is non-verbal.^1^

Usage of personal protective equipment is undoubtedly necessary for prevention of spread of infection from patients to healthcare workers and vice versa. However, the masks, goggles and other headgear make it impossible to even maintain eye contact with patients, let alone effective communication. The spacesuit-like attire creates a rift between the doctor and patient at first sight itself.

Talking through all the headgear has an effect on voice modulation and sometimes one has to shout out loud to get the message across. Intonation of voice to convey appropriate emotions is made impossible by the tight-fitting masks.

Differently abled patients who rely on alternate modes of communication (such as lip-reading) are facing tremendous difficulties. The other day I saw a patient with hearing impairment who found it extra difficult to communicate with me as he could not lip-read as he usually does, and I had to ‘restrain’ myself from the temptation to take my mask off and talk to him.

Almost all non-vocal cues are obliterated by protective headgear. Masks cover the lower part of the face, so you cannot tell whether one is smiling or frowning. If the eyes are not covered, one may ‘smile with the eyes’, but the goggles obscure that too. Whatever remains of the facial expressions are annihilated by the face shields.

The protective body suits affect hand gestures and other facets of body language, especially the maintenance of an appropriate posture.

As physicians we solve problems: often medical and sometimes non-medical too. Doubtlessly, it is the need of the hour to come up with solutions to these issues. However, the safety of the healthcare workers and patients cannot be ignored too.

Some healthcare workers have made attempts to minimise this communication barrier by various means, such as sticking-on smiling pictures of themselves onto their protective bodysuit. Unfortunately, these measures cannot fully replace all the facets of non-verbal communication.

Therefore, it is vital to explore innovative solutions to overcome these issues, as we adapt to a new way of life with the ongoing pandemic.
